# Clinical effectiveness of tigecycline in combination therapy against nosocomial pneumonia caused by CR-GNB in intensive care units: a retrospective multi-centre observational study

**DOI:** 10.1186/s40560-022-00647-y

**Published:** 2023-01-03

**Authors:** Kuang-Yao Yang, Chung-Kan Peng, Chau-Chyun Sheu, Yu-Chao Lin, Ming-Cheng Chan, Sheng-Huei Wang, Chia-Min Chen, Chih-Yu Chen, Zhe-Rong Zheng, Jia-Yih Feng, the T-CARE (Taiwan Critical Care and Infection) Group

**Affiliations:** 1 Department of Chest Medicine, Taipei Veterans General Hospital, #201, Sec. 2, Shih-Pai Road, Taipei, 11217 Taiwan; 2 Institute of Emergency and Critical Care Medicine, School of Medicine, National Yang Ming Chiao Tung University, Taipei, Taiwan; 3 Cancer Progression Research Center, National Yang Ming Chiao Tung University, Taipei, Taiwan; 4 Division of Pulmonary and Critical Care Medicine, Department of Internal Medicine, Tri-Service General Hospital, National Defense Medical Center, Taipei, Taiwan; 5 Division of Pulmonary and Critical Care Medicine, Department of Internal Medicine, Kaohsiung Medical University Hospital, Kaohsiung Medical University, Kaohsiung, Taiwan; 6 Department of Internal Medicine, School of Medicine, College of Medicine, Kaohsiung Medical University, Kaohsiung, Taiwan; 7 Division of Pulmonary and Critical Care Medicine, Department of Internal Medicine, China Medical University Hospital, Taichung, Taiwan; 8 School of Medicine, China Medical University, Taichung, Taiwan; 9 Department of Critical Care Medicine, Taichung Veterans General Hospital, Taichung, Taiwan; 10 School of Post Baccalaureate Medicine, National Chung Hsing University, Taichung, Taiwan; 11 Graduate Institute of Medical Sciences, National Defense Medical Center, Taipei, Taiwan; 12 Division of Pulmonary Medicine, Department of Internal Medicine, Chung Shan Medical University Hospital, Taichung, Taiwan; 13 Division of Chest Medicine, Department of Internal Medicine, Taichung Veterans General Hospital, Taichung, Taiwan; 14 School of Medicine, National Yang Ming Chiao Tung University, Taipei, Taiwan

**Keywords:** Nosocomial pneumonia, Tigecycline, Carbapenem-resistant Gram-negative bacteria, Clinical failure, Mortality

## Abstract

**Background:**

Tigecycline has in vitro bacteriostatic activity against a broad spectrum of bacteria, including carbapenem-resistant Gram-negative bacteria (CR-GNB). However, the role of tigecycline in treatment of nosocomial pneumonia caused by CR-GNB remains controversial and clinical evidences are limited. We aimed to investigate the clinical benefits of tigecycline as part of the combination treatment of nosocomial CR-GNB pneumonia in intensive care unit (ICU).

**Methods:**

This multi-centre cohort study retrospectively enrolled ICU-admitted patients with nosocomial pneumonia caused by CR-GNB. Patients were categorized based on whether add-on tigecycline was used in combination with at least one anti-CR-GNB antibiotic. Clinical outcomes and all-cause mortality between patients with and without tigecycline were compared in the original and propensity score (PS)-matched cohorts. A subgroup analysis was also performed to explore the differences of clinical efficacies of add-on tigecycline treatment when combined with various anti-CR-GNB agents.

**Results:**

We analysed 395 patients with CR-GNB nosocomial pneumonia, of whom 148 received tigecycline and 247 did not. More than 80% of the enrolled patients were infected by CR-*Acinetobacter baumannii* (CRAB). A trend of lower all-cause mortality on day 28 was noted in tigecycline group in the original cohort (27.7% vs. 36.0%, *p* = 0.088). In PS-matched cohort (102 patient pairs), patients with tigecycline had significantly lower clinical failure (46.1% vs. 62.7%, *p* = 0.017) and mortality rates (28.4% vs. 52.9%, *p* < 0.001) on day 28. In multivariate analysis, tigecycline treatment was a protective factor against clinical failure (PS-matched cohort: aOR 0.52, 95% CI 0.28–0.95) and all-cause mortality (original cohort: aHR 0.69, 95% CI 0.47–0.99; PS-matched cohort: aHR 0.47, 95% CI 0.30–0.74) at 28 days. Kaplan–Meier survival analysis in subgroups of patients suggested significant clinical benefits of tigecycline when added to a colistin-included (log rank *p* value 0.005) and carbapenem-included (log rank *p* value 0.007) combination regimen.

**Conclusions:**

In this retrospective observational study that included ICU-admitted patients with nosocomial pneumonia caused by tigecycline-susceptible CR-GNB, mostly CRAB, tigecycline as part of a combination treatment regimen was associated with lower clinical failure and all-cause mortality rates.

**Supplementary Information:**

The online version contains supplementary material available at 10.1186/s40560-022-00647-y.

## Background

Nosocomial pneumonia, including hospital-acquired pneumonia (HAP) and ventilator-associated pneumonia (VAP), are the leading cause of morbidity and mortality in the intensive care unit (ICU) [1, 2]. Among the various pathogens that cause nosocomial pneumonia, a focus on carbapenem-resistant Gram-negative bacteria (CR-GNB), especially CR-*Acinetobacter baumannii complex* (CRAB), CR-*Enterobacteriaceae* (CRE), and CR-*Pseudomonas aeruginosa* (CRPA)*,* is motivated by the limited number of treatment choices available and poor treatment outcomes [3, 4]. According to the latest guidelines regarding multidrug-resistant organisms (MDRO), novel β-lactam/β-lactamase inhibitors, especially ceftazidime–avibactam, are currently the treatment of choices for CRE and CRPA [5–7]. However, clinical isolates with resistance to novel β-lactam/β-lactamase inhibitors are emerging [8]. For CRE with resistance to novel agents and for CRAB, combination therapy of old drugs, including polymyxin, tigecycline, minocycline, and aminoglycoside, should be considered, especially in those with moderate-to-high disease severity [5–7]. However, the optimal combination regimen of antibiotics remains uncertain.

Tigecycline has in vitro bacteriostatic activity against a broad spectrum of drug-resistant bacteria [9, 10]. However, tigecycline has a sub-optimal concentration in epithelial lining fluid, blood, and urine [11]. Previous randomized controlled trials failed to prove the efficacy of tigecycline in the treatment of nosocomial pneumonia [12]. Although recent HAP/VAP guidelines recommended against the use of tigecycline in nosocomial caused by CR-GNB [4], tigecycline is frequently used off-label due to the limited antibiotic choices for nosocomial pneumonia caused by CR-GNB. The latest MDRO guidelines recommended including high-dose tigecycline in combination regimen against CRE and CRAB [5–7]. Several previous observational studies demonstrated the potential of tigecycline to improve clinical response and decrease mortality in patients with nosocomial pneumonia. However, most of them are limited by a small sample size and significant heterogeneity in treatment strategies used [13–16].

Nosocomial pneumonia caused by CR-GNB is an emerging problem with limited antibiotic treatment options. Tigecycline has in vitro bacteriostatic activity against CR-GNB, but the clinical benefits of add-on tigecycline as part of a combination regimen CR-GNB-related nosocomial pneumonia remains uncertain. In the present study, we compared the clinical response rate and mortality rate between patients with HAP/VAP caused by CR-GNB who received add-on tigecycline treatment and those who did not. We hypothesized that add-on tigecycline as part of the combination regimen would provide clinical benefits. A subgroup analysis was also performed in order to determine whether the proposed synergistic benefits of add-on tigecycline treatment are dependent on other antibiotics used to treat nosocomial pneumonia caused by CR-GNB.

## Methods

### Patients and study setting

This was a multi-centre retrospective cohort study conducted at five referral medical centres in Taiwan between January 2016 and December 2016. The major aim of this study was to investigate the impact of antibiotics regimens on treatment outcomes of patients with HAP and VAP caused by CR-GNB. The study design and relevant prior analyses have been described [14, 17]. The eligibility criteria for inclusion in this study were as follows: (1) ICU-admitted patients diagnosed with HAP/VAP, which developed more than 48 h after admission; (2) positive cultures for CR-GNB, which is resistant to at least one of the carbapenems, were identified from respiratory specimens; and (3) received at least one key parenteral antibiotic considered for the treatment of pneumonia caused by CR-GNB, including colistin, sulbactam (ampicillin–sulbactam or cefoperazone–sulbactam), aminoglycoside, and carbapenem. The exclusion criteria were as follows: (1) age < 20 years old; (2) diagnosis of community-acquired pneumonia, healthcare associated pneumonia (HCAP), or concomitant lung cancer with obstructive pneumonitis; (3) CR-GNB showed resistance to tigecycline; (4) positive culture of *Pseudomonas aeruginosa*; (5) intravenous tigecycline with duration < 2 days and/or daily dosage < 100 mg.

The study protocol was approved by the Institutional Review Board of all the participating hospitals (2018-03-001CC, 1-107-05-054, CE18100A, CMUH107-REC3-052, and KMUHIRB-E(I)-20180141). The need for informed consents was waived.

### Data collection and disease severities definitions

Demographic characteristics and underlying comorbidities were retrospectively collected from complete electronic patient files from participating hospitals. Disease severity was evaluated by Acute Physiology and Chronic Health Evaluation (APACHE) II score on ICU-admission day; Sequential Organ Failure Assessment (SOFA) scores on ICU-admission day and the pneumonia index date; and presence of organ dysfunction [including septic shock (vasopressor use), renal failure (under dialysis), and respiratory failure (with mechanical ventilator and PF ratio < 200)] upon pneumonia diagnosis.

### Pneumonia definitions

HAP refers to pneumonia occurring ≥ 48 h after hospital admission, and VAP refers to pneumonia developing ≥ 48 h after endotracheal intubation with an invasive mechanical ventilator. Causative organisms were defined as CR-GNB that were isolated from respiratory specimens, including sputum, endotracheal aspirates, bronchoalveolar lavage fluid with a concentration of ≥ 10^4^ colony-forming units (CFU)/mL, and protected specimen brush with a concentration of ≥ 10^3^ CFU/mL. For sputum and endotracheal aspirate, moderate-to-heavy growth by semi-quantitative method was considered to have HAP/VAP. The index culture study collection date was defined as the pneumonia index date. Definition of HCAP is provided in materials and methods of Additional file 1.

### Microbiological tests and resistance determination

The results of susceptibility tests to carbapenems were determined according to the Clinical and Laboratory Standards Institute (CLSI) recommendations, 30th edition [18]. Carbapenem resistance was defined as resistance to imipenem or meropenem (imipenem or meropenem MIC ≥ 4 mg/L for Enterobacterales and MIC ≥ 8 mg/L for *Acinetobacter* spp.) Susceptibilities of tigecycline were determined according to the FDA standard (MIC ≤ 2 mg/L, sensitive; MIC = 4 mg/L, intermediate; MIC ≥ 8 mg/L, resistant) [19].

### Treatment regimens and outcomes evaluation

Intravenous antibiotics that were used during the treatment course of nosocomial pneumonia with a duration ≥ 2 days were recorded. The daily dosage and treatment duration of intravenous tigecycline with a duration ≥ 2 days were recorded specifically for further analysis. Novel β-lactam/β-lactamase inhibitors, such as ceftazidime–avibactam and ceftolozane–tazobactam, were not available in Taiwan during the study period.

All the patients were followed until discharge or death. Treatment outcomes were compared between patients with and without add-on tigecycline in an antibiotic regimen. The outcomes evaluated in the present study included the clinical response rate, assessed on days 7, 14, and 28, and the all-cause mortality rate, assessed on days 14, 28, and upon discharge. Clinical responses were classified as “success” (resolution or substantial improvement of symptoms/signs of pneumonia, improvement or lack of progression of chest radiographic abnormalities, and no additional antibacterial therapy was required or was antibiotics free) and “failure” (no apparent response to therapy, persistent or worsening of symptoms/signs of pneumonia, persistent or progression of radiographic abnormalities that required additional antibiotic therapy, or death). Other outcomes of interest included ventilator weaning, new-onset dialysis, ICU stays, and hospital stays. All patients were followed up until death or hospital discharge. Details of clinical outcomes evaluation are provided in materials and methods of Additional file 1.

### Time-window bias adjustment and propensity score matching

In considering the time-window bias related to delayed initiation of tigecycline, and possible differences in demographic characteristics and disease severity between patients with and without add-on tigecycline [20], we created a second cohort after time-window bias adjustment and propensity score (PS) matching. For time-window bias adjustment, patients who died within 3 days of the onset of pneumonia, or who were started on tigecycline more than 3 days of the pneumonia index date, were excluded. After time-window bias adjustment, a PS-matched cohort was built with a propensity score (PS) approach with 1:1 matching and calliper width of 0.2 applied to both patients with and without tigecycline treatment [21]. Propensity scores were created through a logistic regression as a function of age, sex, smoking, pathogens, pneumonia types, comorbidities, APACHE II scores (ICU admission), SOFA scores (pneumonia index date), albumin levels (pneumonia index date), presence of organs failure, and the key intravenous antibiotic used against CR-GNB.

### Subgroup analysis of tigecycline-containing regimen

A subgroup analysis was performed to explore the differences of clinical efficacies of add-on tigecycline treatment when combined with various anti-CR-GNB agents, including colistin, carbapenem, and sulbactam. Patients were categorized as colistin group if intravenous colistin were included in the regimen, irrespective of the usage of other antibiotics. Patients were categorized as carbapenem groups or sulbactam group following the same rule. The treatment outcomes between patients with and without add-on tigecycline were compared in each subgroup of patients.

### Statistical analysis

Statistical analyses were performed using the SPSS version 25.0 software (SPSS, Inc., Chicago, IL, USA). The clinical outcomes and mortality rates of patients with and without tigecycline add-on treatment were compared, using the Mann–Whitney U test for continuous numerical data, and the Pearson’s Chi-squared or Fisher’s exact tests for categorical data, respectively. Multiple imputation was used to compensate for with mean values was used for missing data. In a subgroup analysis, we further compared patients stratified according to the specific anti-CR-GNB antibiotic used. Kaplan–Meier survival curves were constructed to evaluate differences in all-cause mortality between the two groups. A stepwise Cox proportional hazard regression analysis was performed to identify the independent variables associated with mortality at day 28. Binary logistic regression analysis with forward stepwise selection was performed to determine the independent variables associated with clinical failure at day 28. All variables with a *p*-value < 0.1 at the univariate level were included in the multivariate model. All tests were two-tailed and a *p*-value < 0.05 was considered statistically significant.

## Results

### Patient characteristics

**Fig. 1:**
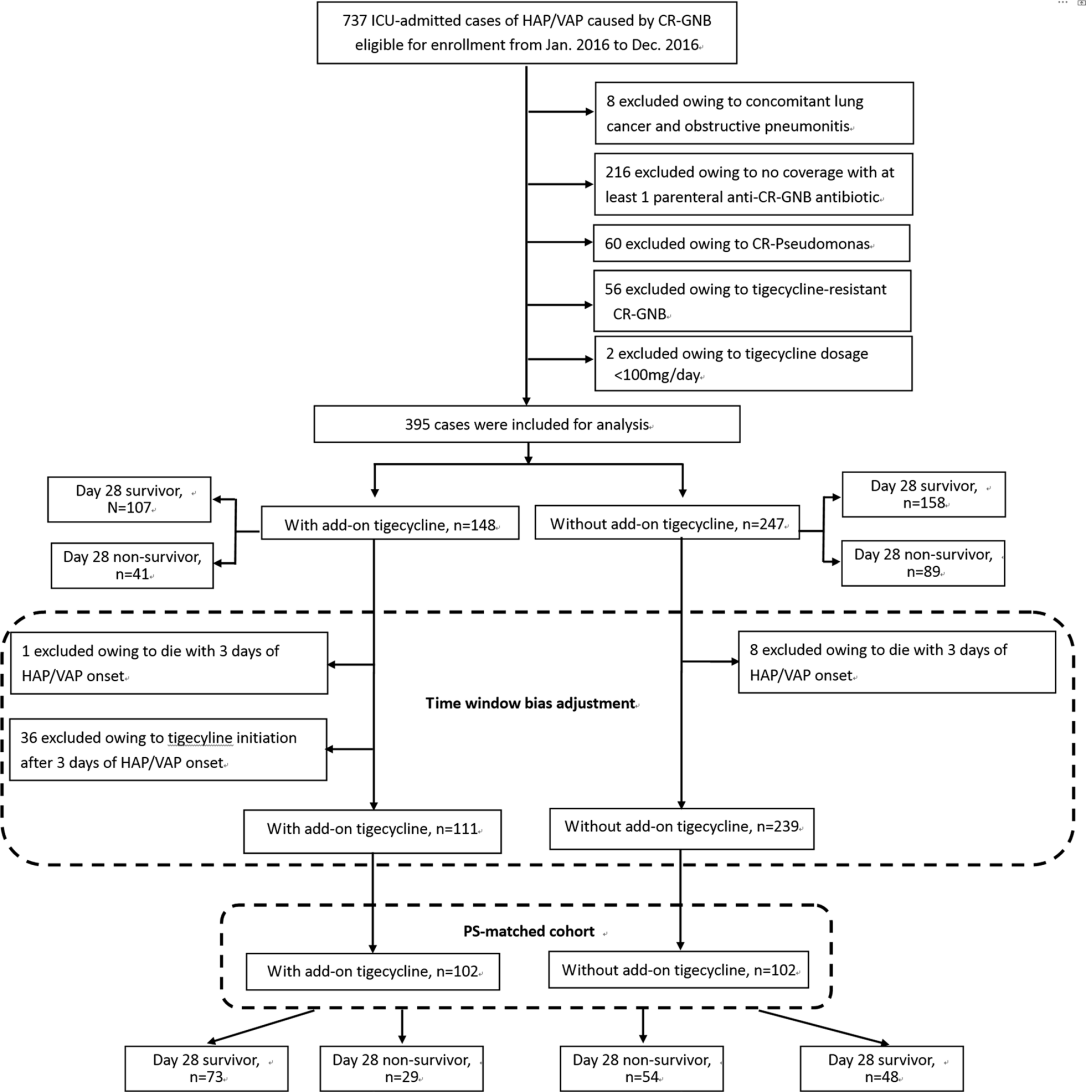
Study flow diagram and patient exclusion criteria. *CR-GNB* carbapenem-resistant Gram-negative bacteria, *HAP* hospital-acquired pneumonia, *VAP* ventilator-associated pneumonia, *ICU* intensive care unit

The present sample was selected from a group of 737 patients with nosocomial pneumonia caused by CR-GNB who were admitted to the ICU between January 2016 and December 2016. A flow diagram showing the numbers of cases and reasons for exclusion is shown in Fig. 1. In total, 395 cases fulfilled the inclusion criteria of whom 148 received add-on tigecycline treatment and 247 did not. As summarized in Table 1, nosocomial pneumonia was primarily caused by CRAB (81.3%), and 71.1% of cases were admitted to the medical ICU. The median APACHE II score upon ICU admission was 23 (18–27) and the median SOFA score on nosocomial pneumonia onset was 8 (5–10).

**Table 1:** Demographic characteristics and disease severities of ICU patients with nosocomial pneumonia treated with and without add-on tigecycline in combination regimens

	All cases	Add-on tigecycline		
		Yes	No	
Case number	395	148	247	
Mean age (SD)	70.0 (15.7)	70.6 (13.6)	69.7 (16.9)	0.552
Male	261 (66.1%)	98 (66.2%)	163 (66.0%)	0.964
Mean BMI (SD)	23.0 (4.5)	23.2 (4.9)	22.8 (4.2)	0.347
Smoking history	151 (38.2%)	59 (39.9%)	92 (37.2%)	0.604
Alcohol consumption	67 (17.0%)	27 (18.2%)	40 (16.2%)	0.599
Isolated pathogens				0.087
CRAB	321 (81.3%)	128 (86.5%)	193 (78.1%)	
CRE	74 (18.7%)	20 (13.6%)	54 (21.8%)	
Pneumonia types				0.574
HAP	116 (29.4%)	41 (27.7%)	75 (30.4%)	
VAP	279 (70.6%)	107 (72.3%)	172 (69.6%)	
ICU types				0.190
Medical ICU	281 (71.1%)	111 (75.0%)	170 (68.8%)	
Surgical ICU	114 (28.9%)	37 (25.0%)	77 (31.2%)	
Comorbidities				
Malignancies	54 (13.7%)	16 (10.8%)	38 (15.4%)	0.200
Renal insufficiency	39 (9.9%)	15 (10.1%)	24 (9.7%)	0.893
Chronic lung diseases	71 (18.0%)	28 (18.9%)	43 (17.4%)	0.705
Diabetes	139 (35.2%)	59 (39.9%)	80 (32.4%)	0.132
Autoimmune disease	18 (4.6%)	10 (6.8%)	8 (3.2%)	0.105
Intravenous antibiotics				
Colistin	167 (42.3%)	64 (43.2%)	103 (41.7%)	0.764
Sulbactam	138 (34.9%)	28 (18.9%)	110 (44.5%)	< 0.001
Carbapenem	156 (39.5%)	58 (39.2%)	98 (39.7%)	0.924
Antibiotics susceptibility				
Sulbactam resistance	99 (25.1%)	47 (31.8%)	52 (21.1%)	0.017
Colistin resistance	3 (0.8%)	2 (1.4%)	1 (0.4%)	0.563
APACHE II scores^a^ (median, IQR)	23 (18–27)	22 (17–27)	23 (18–27)	0.098
SOFA scores (median, IQR)				
ICU admission	8 (5–10)	8 (6–11)	7 (5–10)	0.013
Pneumonia index date	8 (5–10)	8 (6–11)	7 (5–10)	0.090
Presenting features^b^				
Septic shock	65 (16.5%)	29 (19.6%)	36 (14.6%)	0.193
Invasive ventilator	364 (92.2%)	141 (95.3%)	223 (90.3%)	0.074
PF ratio < 200	109 (27.6%)	38 (25.7%)	71 (28.7%)	0.509
Dialysis^c^	47 (11.9%)	18 (12.2%)	29 (11.7%)	0.900
Laboratory results^d^ (median, IQR)				
Leukocytes (× 10^9^ per L)	12.5 (8.3–16.6)	12.8 (8.7–18.6)	12.3 (8.2–16.1)	0.194
Albumin (g/dL)	2.7 (2.3–3.0)	2.6 (2.2–2.9)	2.7 (2.4–3.1)	< 0.001
CRP (mg/dL)	8.6 (5.1–15.0)	8.6 (5.6–15.7)	8.6 (4.9–14.8)	0.500
Tigecycline (median, IQR)				
Daily dosage				
200 mg	6 (4.1%)	6 (4.1%)	–	–
100 mg	142 (95.9%)	142 (95.9%)	–	–
Treatment duration (days)	–	7 (6–14)	–	–

Data are presented as *n* (%)

*APACHE II* Acute Physiology and Chronic Health Evaluation II, *BMI* body mass index, *CRP* carbapenem-*resistant Pseudomonas aeruginosa*, *CRAB* carbapenem-resistant *Acinetobacter baumannii*, *CRE* carbapenem-resistant Enterobacteriaceae, *HAP* hospital-acquired pneumonia, *ICU* intensive care unit, *IQR* interquartile range, *PF ratio* PaO_2_/FiO_2_ ratio, *SD* standard deviation, *SOFA* Sequential Organ Failure Assessment, *VAP* ventilator-associated pneumonia

^a^Obtained on ICU admission date

^b^Presence of organ dysfunction on pneumonia index date

^c^Including hemodialysis and continuous venovenous hemofiltration

^d^Obtained on pneumonia index date

Patients who received add-on tigecycline treatment were less likely to receive sulbactam, had higher SOFA scores upon ICU admission, and had lower serum albumin levels on pneumonia index date. Patients with add-on tigecycline also showed a trend towards a higher proportion of nosocomial pneumonia caused by CRAB, and a trend towards more patients having received invasive ventilator support when nosocomial pneumonia occurred. A majority of the patients received tigecycline with daily dosage of 100 mg and the median duration of tigecycline treatment was 7 days (6–14 days). The age, sex, and underlying diseases were comparable between patients with and without add-on tigecycline.

### PS-matched cohort after time-window bias adjustment

After time-window bias adjustment and PS matching (Fig. 1), we built a PS-matched cohort that included 102 patient pairs. As shown in Table 2, there were no significant differences in demographic characteristics, underlying comorbidities, disease severity, antibiotics used, or laboratory results between patients stratified according to add-on tigecycline treatment in the PS-matched cohort.

**Table 2:** Demographic characteristics and disease severities of propensity score-matched ICU patients with nosocomial pneumonia treated with and without add-on tigecycline in combination regimen

	Add-on tigecycline		*p* value
	Yes	No	
Case number	102	102	
Mean age (SD)	71.2 (13.2)	70.7 (16.3)	0.806
Male	63 (61.8%)	67 (65.7%)	0.560
Mean BMI (SD)	22.7 (4.7)	22.4 (3.9)	0.614
Smoking history	37 (36.3%)	38 (37.3%)	0.885
Alcohol consumption	15 (14.7%)	14 (13.7%)	0.841
Isolated pathogens			0.845
CRAB	87 (85.3%)	86 (84.3%)	
CRE	15 (14.7%)	16 (15.7%)	
Pneumonia types			0.756
HAP	30 (29.4%)	28 (27.5%)	
VAP	72 (70.6%)	74 (72.5%)	
ICU types			0.219
Medical ICU	76 (74.5%)	68 (66.7%)	
Surgical ICU	26 (25.5%)	34 (33.3%)	
Comorbidities			
Malignancies	16 (15.7%)	16 (15.7%)	1.000
Renal insufficiency	10 (9.8%)	15 (14.7%)	0.286
Chronic lung diseases	19 (18.6%)	18 (17.6%)	0.856
Diabetes	39 (38.2%)	40 (39.2%)	0.886
Autoimmune disease	7 (6.9%)	7 (6.9%)	1.000
Intravenous antibiotics			
Colistin	49 (48.0%)	55 (53.9%)	0.401
Sulbactam	28 (27.5%)	28 (27.5%)	1.000
Carbapenem	37 (36.3%)	39 (38.2%)	0.772
Antibiotics susceptibility			
Colistin resistance	2 (2.2%)	0	0.497
Sulbactam resistance	27 (26.5%)	29 (28.4%)	0.754
APACHE II scores (median, IQR)^a^	23 (17–28)	23 (17–27)	0.945
SOFA scores (median, IQR)			
ICU admission	8 (6–10)	8 (6–11)	0.506
Pneumonia index date	8 (6–11)	8 (6–11)	0.319
Presenting features^b^			
Septic shock	16 (15.7%)	18 (17.6%)	0.707
Invasive ventilator	98 (96.1%)	91 (89.2%)	0.097
PF ratio < 200	32 (31.4%)	34 (33.3%)	0.765
Dialysis^c^	12 (11.8%)	14 (13.7%)	0.675
Laboratory results (median, IQR)^d^			
Leukocytes (× 10^9^ per L)	12.7 (8.2–17.4)	11.2 (6.2–16.0)	0.161
Albumin (g/dL)	2.6 (2.3–2.8)	2.7 (2.3–3.1)	0.051
CRP (mg/dL)	8.6 (5.6–14.3)	8.8 (6.3–15.4)	0.531
Tigecycline			–
Daily dosage			
200 mg	4 (5.1%)	–	–
100 mg	75 (94.9%)	–	–
Treatment duration (days)	7 (6–14)	–	–

Data are presented as *n* (%)

*APACHE II* Acute Physiology and Chronic Health Evaluation II, *BMI* body mass index, *CRP* carbapenem-resistant* Pseudomonas aeruginosa*, *CRAB* carbapenem-resistant *Acinetobacter baumannii*, *CRE* carbapenem-resistant Enterobacteriaceae, *HAP* hospital-acquired pneumonia, *ICU* intensive care unit, *IQR* interquartile range, *IV* intravenous, *PF ratio* PaO_2_/FiO_2_ ratio, *SD* standard deviation, *SOFA* Sequential Organ Failure Assessment, *VAP* ventilator-associated pneumonia

^a^Obtained on ICU admission date

^b^Presence of organ dysfunction on pneumonia index date

^c^Including hemodialysis and continuous venovenous hemofiltration

^d^Obtained on pneumonia index date

### Add-on tigecycline was associated with better treatment outcomes

Treatment outcomes of patients with nosocomial pneumonia with and without tigecycline add-on treatment are shown in Table 3. In the original cohort, patients who received add-on tigecycline treatment had a trend towards a lower mortality rate on day 28 (27.7% vs. 36%, *p* = 0.088), and a lower clinical failure rate on day 14 (39.2% vs. 47.8%, *p* = 0.097) compared to patients without add-on tigecycline. The clinical failure rate on days 7 and 28, hospital mortality rate, and 28-day ventilator weaning rate were comparable between the two groups.

**Table 3:** Treatment outcomes of propensity score-matched ICU patients with nosocomial pneumonia treated with and without add-on tigecycline in combination regimen

	Original cohort			PS-matched cohort		
	With add-on tigecycline	Without add-on tigecycline	*P* value	With add-on tigecycline	Without add-on tigecycline	*P* value
Case number	148	247		102	102	
Clinical failure						
Day 7	64 (43.2%)	103 (41.7%)	0.764	38 (37.3%)	53 (52.0%)	0.035
Day 14	58 (39.2%)	118 (47.8%)	0.097	40 (39.2%)	59 (57.8%)	0.008
Day 28	67 (45.3%)	121 (49.0%)	0.474	47 (46.1%)	64 (62.7%)	0.017
All-cause mortality						
Day 28	41 (27.7%)	89 (36.0%)	0.088	29 (28.4%)	54 (52.9%)	< 0.001
Hospital mortality	74 (50.0%)	120 (48.6%)	0.785	54 (52.9%)	70 (68.6%)	0.022
28-day ventilator weaning^a^	66/144 (45.8%)	105/223 (47.1%)	0.814	41/100 (41.0%)	29/91 (31.9%)	0.191
Newly onset dialysis^b^	11 (7.4%)	23 (9.3%)	0.519	9 (8.8%)	13 (12.7%)	0.367
ICU stays (median, IQR) (days)	27 (17–46)	23 (15–40)	0.386	27.5 (18–44)	24.5 (16–42)	0.931
Hospital stays (median, IQR) (days)	51 (35–79)	46 (30–68)	0.268	51 (37–80)	41.5 (26–68)	0.827

Data are presented as *n* (%)

^a^Only cases with invasive ventilator were included for analysis

^b^Including hemodialysis and continuous venovenous hemofiltration within 28 day

In the PS-matched cohort, patients who received add-on tigecycline treatment had significantly lower clinical failure rates on day 7 (37.3% vs. 52.0%, *p* = 0.035), 14 (39.2% vs. 57.8%, *p* = 0.008), and day 28 (46.1% vs. 62.7%, *p* = 0.017), and lower mortality rates on day 28 (28.4% vs. 52.9%, *p* < 0.001), and a lower hospital mortality rate (52.9% vs. 68.6%, *p* = 0.022).

**Fig. 2:**
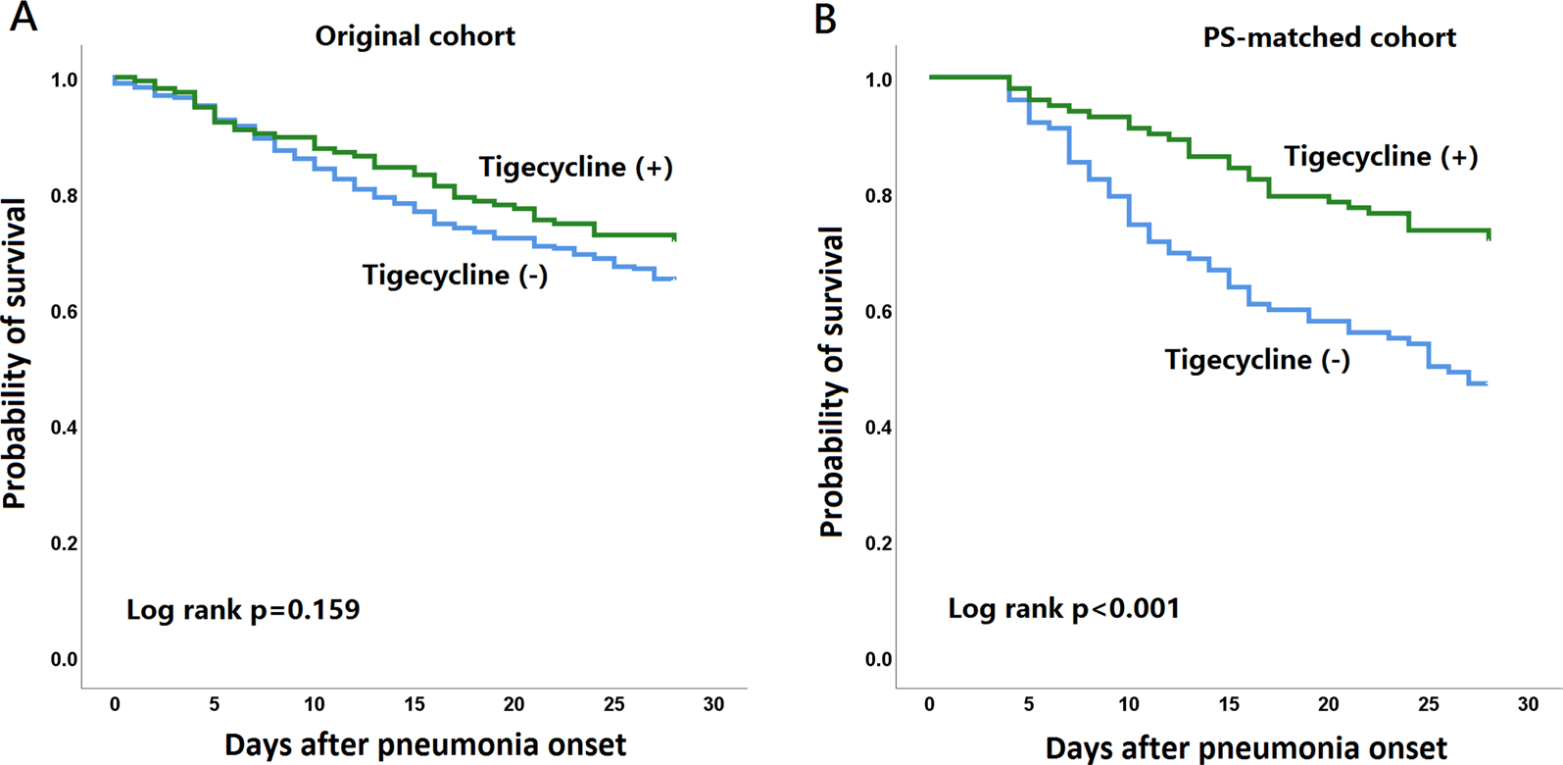
Kaplan–Meier analysis of 28-day mortality status between patients with and without add-on tigecycline treatment in the **A** original cohort and **B** PS-matched cohort

Kaplan–Meier analyses of all-cause mortalities in the original cohort and PS-matched cohort are shown in Fig. 2. In the original cohort, there was no significant difference in 28-day mortality between patients who received add-on tigecycline treatment and those who did not (Fig. 2A). In the PS-matched cohort, patients who received add-on tigecycline treatment had a lower 28-day mortality risk compared to patients without add-on tigecycline (Fig. 2B). The curves separated early after pneumonia onset.

### Independent factors associated with treatment outcomes

Univariate and multivariate analyses were performed to identify independent clinical factors associated with all-cause mortality and clinical failure rates at day 28. In the original cohort, independent factors associated with all-cause mortality on day 28 included body mass index (BMI) (adjusted hazard ratio [aHR] 0.94, 95% confidence interval [CI] 0.90–0.98), SOFA scores on the pneumonia index date (aHR 1.17, 95% CI 1.11–1.24), and tigecycline treatment (aHR 0.69, 95% CI 0.47–0.99) (Table 4). Independent factors associated with clinical failure on day 28 included BMI (adjusted odds ratio [aOR] 0.92, 95% CI 0.88–0.97), pneumonia caused by CRAB (aOR 2.21, 95% CI 1.27–3.87), and SOFA scores on the pneumonia index date (aOR 1.20, 95% CI 1.11–1.29). In the PS-matched cohort, independent factors associated with all-cause mortality on day 28 included BMI (aHR 0.93, 95% CI 0.88–0.99), SOFA scores on the pneumonia index date (aHR 1.12, 95% CI 1.06–1.19), and tigecycline treatment (aHR 0.47, 95% CI 0.30–0.74) (Table 5). Independent factors associated with clinical failure on day 28 included BMI (aHR 0.90, 95% CI 0.83–0.97), SOFA scores on the pneumonia index date (aOR 1.19, 95% CI 1.09–1.31), and tigecycline treatment (aOR 0.52, 95% CI 0.28–0.95).

**Table 4:** Univariate and multivariate analysis of clinical factors associated with 28-day mortality and day 28 clinical failure in ICU patients with nosocomial pneumonia caused by CR-GNB

	28-day mortality^a^				Day 28 clinical failure^b^			
	Univariate analysis		Multivariate analysis		Univariate analysis		Multivariate analysis	
	HR (95% CI)	*P* value	aHR (95% CI)	*P* value	OR (95% CI)	*P* value	aOR (95% CI)	*P* value
Age	1.00 (0.99–1.01)	0.843			1.01 (1.00–1.02)	0.184		
Male	0.97 (0.68–1.40)	0.880			0.90 (0.60–1.37)	0.636		
BMI	0.93 (0.90–0.97)	0.001	0.94 (0.90–0.98)	0.006	0.92 (0.88–0.97)	0.001	0.92 (0.88–0.97)	0.002
CRAB	0.95 (0.61–1.47)	0.815			1.16 (0.70–1.93)	0.567	2.21 (1.27–3.87)	0.005
Medical ICU	1.27 (0.86–1.89)	0.232			1.44 (0.92–2.23)	0.107		
Malignancies	1.06 (0.64–1.74)	0.830			0.79 (0.44–1.41)	0.429		
Renal insufficiency	1.29 (0.77–2.18)	0.335			1.66 (0.85–3.26)	0.137		
Chronic lung diseases	1.52 (1.02–2.28)	0.042	1.45 (0.96–2.18)	0.075	2.19 (1.29–3.72)	0.004	0.72 (0.46–1.12)	0.146
Diabetes	0.74 (0.51–1.07)	0.109			0.69 (0.46–1.05)	0.086		
APACHE II score^c^	1.01 (0.99–1.03)	0.363			1.02 (0.99–1.04)	0.263		
SOFA score^d^	1.15 (1.10–1.21)	< 0.001	1.17 (1.11–1.24)	< 0.001	1.18 (1.11–1.25)	< 0.001	1.20 (1.11–1.29)	< 0.001
PF ratio ≤ 200^e^	1.36 (0.94–1.97)	0.100			1.44 (0.92–2.24)	0.109		
Septic shock^e^	1.60 (1.06–2.41)	0.027	0.73 (0.46–1.18)	0.206	1.82 (1.06–3.13)	0.030	0.73 (0.37–1.44)	0.367
Dialysis^e^	1.62 (1.03–2.55)	0.036	1.00 (0.61–1.64)	0.996	2.12 (1.13–3.99)	0.019	1.39 (0.69–2.84)	0.359
Albumin ≤ 3 mg/dL^d^	0.99 (0.70–1.39)	0.935			0.95 (0.64–1.41)	0.788		
Sulbactam susceptible	0.96 (0.65–1.43)	0.851			0.56 (0.35–0.9)	0.016	1.53 (0.97–2.40)	0.067
Colistin susceptible	1.41 (0.86–2.29)	0.169			1.42 (0.85–2.37)	0.179		
Add-on tigecycline	0.72 (0.50–1.05)	0.086	0.69 (0.47–0.99)	0.047	0.86 (0.57–1.30)	0.474		

*APACHE II* Acute Physiology and Chronic Health Evaluation II, *CRAB* carbapenem-resistant *Acinetobacter baumannii*, *ICU* intensive care unit, *PF ratio* PaO_2_/FiO_2_ ratio, *SOFA* Sequential Organ Failure Assessment

^a^Adjusted hazard ratio (aHR) and 95% confidence interval (CI) were derived from Cox regression analysis

^b^Adjusted odds ratio (aOR) and 95% CI were derived from logistic regression analysis

^c^Obtaind on ICU admission date

^d^Obtained on pneumonia index date

^e^Presence of organ dysfunction on the pneumonia index date

**Table 5:** Univariate and multivariate analysis of clinical factors associated with 28-day mortality and day 28 clinical failure in propensity score-matched ICU patients with nosocomial pneumonia caused by CR-GNB

	28-day mortality^a^				Day 28 clinical failure^b^			
	Univariate analysis		Multivariate analysis		Univariate analysis		Multivariate analysis	
	HR (95% CI)	*P* value	aHR (95% CI)	*P* value	OR (95% CI)	*P* value	aOR (95% CI)	*P* value
Age	1.00 (0.98–1.01)	0.658			0.74 (0.99–1.03)	0.664		
Male	1.20 (0.76–1.90)	0.438			1.11 (0.63–1.98)	0.642		
BMI	0.93 (0.88–0.98)	0.010	0.93 (0.88–0.99)	0.014	0.90 (0.84–0.96)	0.003	0.90 (0.83–0.97)	0.005
CRAB	0.63 (0.37–1.09)	0.097	0.74 (0.43–1.29)	0.293	1.14 (0.53–2.46)	0.734		
Medical ICU	0.91 (0.57–1.44)	0.683			1.06 (0.58–1.95)	0.842		
Malignancies	1.19 (0.67–2.11)	0.562			1.23 (0.38–1.73)	0.171		
Renal insufficiency	1.06 (0.56–2.00)	0.855			1.13 (0.66–3.74)	0.411		
Chronic lung diseases	1.37 (0.81–2.31)	0.239			1.09 (0.81–3.56)	0.588		
Diabetes	0.70 (0.44–1.10)	0.123			1.45 (0.34–1.07)	0.041	0.68 (0.36–1.26)	0.215
APACHE II score^c^	1.02 (0.99–1.05)	0.194			1.02 (0.98–1.06)	0.348		
SOFA score^d^	1.14 (1.08–1.21)	< 0.001	1.12 (1.06–1.19)	< 0.001	1.19 (1.09–1.30)	< 0.001	1.19 (1.09–1.31)	< 0.001
PF ratio ≤ 200^e^	1.29 (0.83–2.02)	0.263			1.59 (0.88–2.9)	0.128		
Septic shock^e^	1.29 (0.75–2.23)	0.358			1.44 (0.68–3.05)	0.347		
Dialysis^e^	1.25 (0.69–2.26)	0.464			1.69 (0.71–3.99)	0.233		
Albumin ≤ 3 mg/dL^d^	0.99 (0.64–1.53)	0.954			0.84 (0.48–1.46)	0.530		
Sulbactam susceptible	0.94 (0.58–1.52)	0.798			0.57 (0.3–1.08)	0.083	0.66 (0.33–1.31)	0.236
Colistin susceptible	1.02 (0.51–2.05)	0.946			1.20 (0.07–19.38)	0.900		
Add-on tigecycline	0.44 (0.28–0.69)	< 0.001	0.47 (0.30–0.74)	0.001	0.51 (0.29–0.89)	0.017	0.52 (0.28–0.95)	0.032

*APACHE II* Acute Physiology and Chronic Health Evaluation II, *CRAB* carbapenem-resistant *Acinetobacter baumannii*, *ICU* intensive care unit, *PF ratio* PaO_2_/FiO_2_ ratio, *SOFA* Sequential Organ Failure Assessment

^a^Adjusted hazard ratio (aHR) and 95% confidence interval (CI) were derived from Cox regression analysis

^b^Adjusted odds ratio (aOR) and 95%CI were derived from logistic regression analysis

^c^Obtaind on ICU admission date

^d^Obtained on pneumonia index date

^e^Presence of organ dysfunction on the pneumonia index date

### Subgroup analysis

**Fig. 3:**
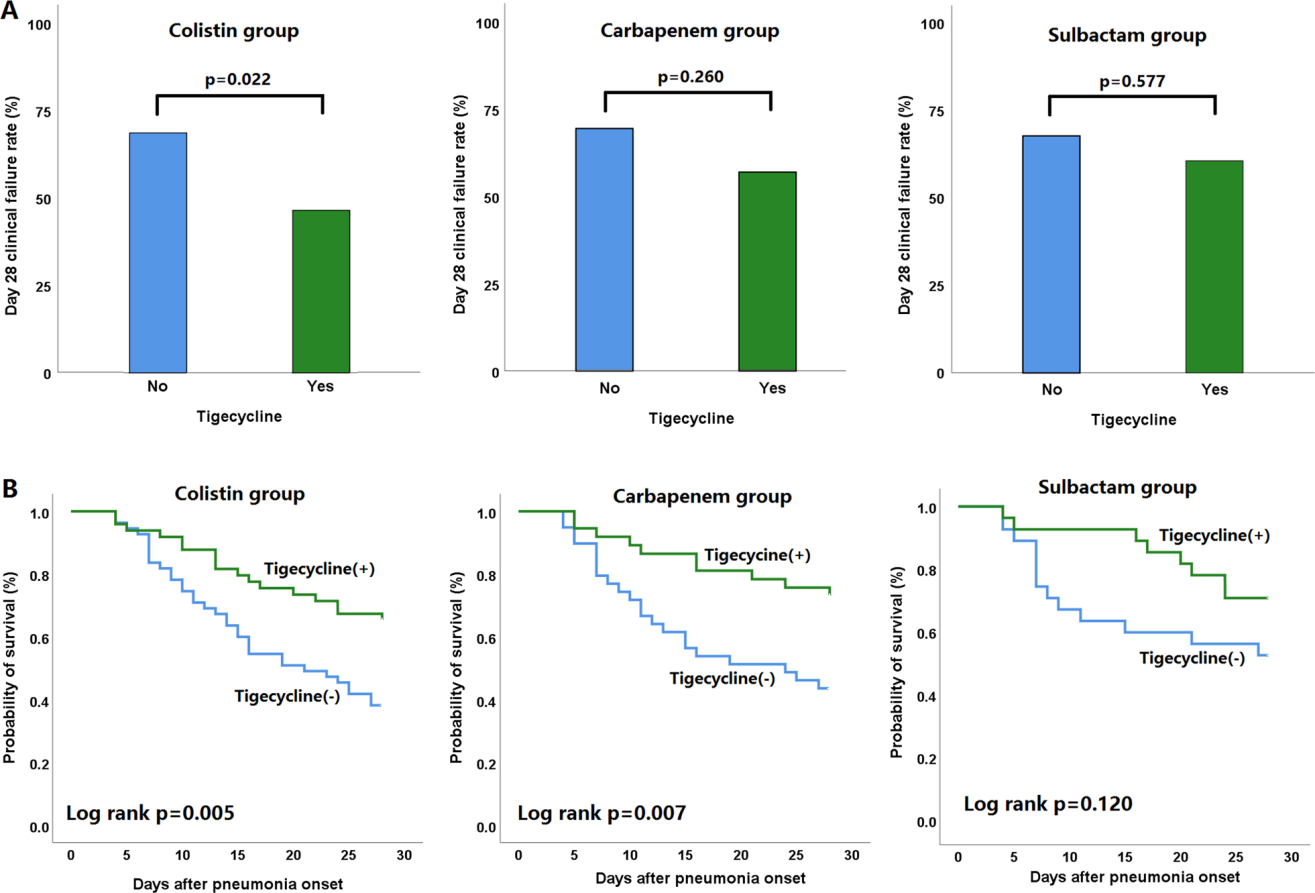
**A** Day 28 clinical failure rate, and **B** Kaplan–Meier analysis of 28-day mortality status between patients with and without add-on tigecycline treatment in subgroups administered with colistin, carbapenem, and sulbactam

We performed a subgroup analysis to identify the clinical effects of add-on tigecycline treatment when combined with colistin, carbapenem, and sulbactam, respectively. As shown in Fig. 3A, add-on tigecycline treatment was significantly associated with a lower clinical failure rate on day 28 in patients treated with colistin, but not in patients treated with carbapenem and sulbactam. In a Kaplan–Meier survival curve analysis, patients who received add-on tigecycline treatment had a lower mortality rate in the subgroup treated with colistin (log rank *p* value 0.005) or carbapenem (log rank *p* value 0.007), but not with sulbactam (Fig. 3B).

Considering that a major portion of the enrolled patients had HAP/VAP caused by CRAB, we made a subgroup analysis in patients infected by CRAB. As shown in Additional file 1: Table S1, in original cohort, patients with add-on tigecycline had lower day 14 clinical failure rate and a trend of lower day 28 mortality rate. After time-window bias adjustment and PS matching, lower clinical failure rate on day 14, day 28, and lower mortality on day 28 were noted in patients with add-on tigecycline.

## Discussion

Nosocomial pneumonia caused by CR-GNB, particularly CRAB, CRE, and CR-pseudomonas, continues to be a growing concern, especially in critically ill patients with ICU admission. When compared with non-CR-GNB pathogens, CR-GNB can significantly increase the risk of mortality in patients with nosocomial infection. Novel β-lactam/β-lactamase inhibitors are the treatment of choice for infection caused by CRE [6, 7], and monotherapy is generally recommended. When novel agents are not available or when CRE isolates are resistant to novel agents, combination of old drugs is suggested [6]. CRAB isolates are generally resistant to novel β-lactam/β-lactamase inhibitors. For moderate to severe infection caused by CRAB, combination of old antibiotics, including sulbactam, polymyxin, tigecycline, minocycline, and aminoglycoside, should be used [5, 6]. When tigecycline is included in a combination regimen against CRE or CRAB, high-dose tigecycline (daily dose 200 mg) is recommended, although the evidence level is low [5–7, 22, 23]

Although tigecycline is recommended as an antibiotic against CRE and CRAB, most evidences came from patients with intra-abdominal infection [24, 25]. The role of tigecycline in nosocomial pneumonia remains controversial. The latest HAP/VAP guideline actually recommended against the use of tigecycline in the treatment of HAP/VAP [4]. Potential complications related to tigecycline, including coagulopathy and pancreatitis, also limited its usage in critically ill patients [26, 27]. A phase III randomized controlled trial reported that tigecycline monotherapy was associated with a higher mortality rate compared with imipenem/cilastatin in patients with HAP [12]. On the other hand, a prospective observational study reported a significantly lower mortality rate for tigecycline/imipenem combination therapy in patients with VAP caused by CRAB, when compared to sulbactam/imipenem combination therapy [28]. Several retrospective observational studies or meta-analyses reported improved responses of tigecycline-containing regimens in patients with nosocomial pneumonia [13, 15, 16, 29], while some studies reported worse outcomes [30, 31]. Some differences in the patient sample between our sample and prior ones bears mentioning. In the present study, all the patients had HAP/VAP that occurred during ICU admission, with a median APACHE II score of 23 and a median SOFA score of 8, which represent a population with a high disease severity. The patients in this study had HAP/VAP caused by tigecycline-susceptible pathogens, and received at least one key anti-CR-GNB agent. Considering the differences in demographic characteristics and disease severities between patients with and without tigecycline, and confounding for survival time bias, we built a PS-matched cohort with survival time bias adjustment. We found that patients with tigecycline in a combination regimen had lower clinical failure rate and all-cause mortality rate on day 28 in PS-matched cohort. In multivariate analysis of PS-matched cohort, we also identified add-on tigecycline as an independent factor associated with lower clinical failure and mortality on day 28. Our findings suggest that add-on tigecycline to a regimen that contains a key anti-CR-GNB antibiotic can further improve the clinical outcomes of critically ill patients with HAP/VAP caused by tigecycline-susceptible CR-GNB.

Our subgroup analysis showed that the clinical benefits of add-on tigecycline were most significant when tigecycline was included as part of a colistin-based regimen, although non-significant trends in favour of a synergistic effect were also evident for carbapenem-based or sulbactam-based regimens. Tigecycline has synergistic effects with colistin, since colistin-induced disruption of the bacterial membrane may facilitate the uptake of tigecycline into the cytoplasm [32]. In vitro synergistic effects between tigecycline and sulbactam or carbapenem have also been reported [33, 34]. The superior treatment responses of tigecycline when used in a colistin-based regimen deserve further validation.

This study had several limitations. First, significant differences in disease severity existed between patients who received add-on tigecycline treatment and those who did not. To address this issue, we performed time-window bias adjustment and PS-matched analysis, and confirmed the clinical benefits of add-on tigecycline in the PS-matched cohort. Second, novel β-lactam/β-lactamase inhibitors were not available during the study period. The synergism between tigecycline and novel agents was therefore not explored. Third, only few of the included patients received high-dose tigecycline. Therefore, the dosage issue of tigecycline could not be further investigated. The benefits of add-on tigecycline in the present study may also be underestimated. Information regarding the adverse events related to tigecycline was not collected and was not reported in the present study. Finally, more than 80% of the enrolled patients had nosocomial pneumonia caused by CRAB. Therefore, the implication of our findings relevant to CRE should be interpreted with caution.

## Conclusions

In this retrospective observational study that included ICU-admitted patients with nosocomial pneumonia caused by CR-GNB, mostly CRAB, tigecycline as part of a combination treatment regimen was associated with lower clinical failure and all-cause mortality rates. Considering the worse treatment outcomes in patients with nosocomial pneumonia, and limited antibiotic choices against CR-GNB, tigecycline could be included as part of a combination antibiotics regimen if microbiological susceptibility is demonstrated. Further prospective controlled trials are warranted to verify our findings and clarify the optimal combination regimen with tigecycline to improve outcomes in eligible patients.

## Supplementary Information

**Additional file 1.** Materials andmethods. **Table S1.** Treatment outcomes of Propensity Score-matched ICU patients with nosocomial pneumonia caused by CRAB treated with and without add-on tigecycline in combination regimen^a^.

## Data Availability

The datasets generated during and/or analysed during the current study are available from the corresponding author on reasonable request.
