# Supplementary material for: Clinical effectiveness of tigecycline in combination therapy against nosocomial pneumonia caused by CR-GNB in intensive care units: a retrospective multi-centre observational study
Source: J Intensive Care. 2023 Jan 3;11:1. doi: 10.1186/s40560-022-00647-y (PMC9808925; doi:10.1186/s40560-022-00647-y)
Supplement: Supplementary file 1 — Additional file 1. Materials andmethods. Table S1. Treatment outcomes of Propensity Score-matched ICU patients with nosocomial pneumonia caused by CRAB treated with and without add-on tigecycline in combination regimena. [file 40560_2022_647_MOESM1_ESM.doc]

**Additional file 1**

**Materials and methods**

*Diagnosis of pneumonia*

The diagnosis of pneumonia was based on new or progressive infiltrates in the chest radiograph, in addition to at least two clinical findings suggestive of pneumonia. The suggestive clinical findings included exacerbated cough, increased production of purulent sputum, fever (≥ 38^o^C) or hypothermia (<35^o^C), and leukocytosis (white cell count ≥ 10000/cumm) or leukopenia (white cell count <4000/cumm). HAP was defined as pneumonia occurring ≥ 48 hours after hospital admission.

Patients presenting with pneumonia diagnosed within 48 hours after hospitalization were considered as healthcare-associated pneumonia (HCAP) if at least one of the following criteria was satisfied: (1) receiving regular dialysis at an outpatient clinic; (2) receiving radiation therapy or chemotherapy at an outpatient clinic; (3) undergoing repeated hospitalization within 90 days prior to the episode of current pneumonia; or (4) residing in a nursing home. Patients with HCAP were excluded from our analysis.

*Clinical outcomes evaluation*

Clinical responses were evaluated on day 7, 14, and 28, and were classified as clinical success and clinical failure. Patients were considered clinical success if the following criteria were met: resolution or substantial improvement of baseline signs and symptoms of pneumonia (leukocytosis, fever, purulent sputum production), an improvement in PF ratio, improvement or lack of progression of chest radiographic abnormalities, no additional antibacterial therapy was required for the treatment of the current infection or was antibiotics free. Patients were considered clinical failure if the following criteria were met: no apparent response to therapy, persistent or worsening of signs/symptoms of pneumonia, no improvement PF ratio, progression of radiographic abnormalities that required additional antibiotic therapy, or death. Clinical information used to determine clinical outcomes was obtained from electric medical records in the study hospitals.

**Table S1.** Treatment outcomes of Propensity Score-matched ICU patients with nosocomial pneumonia caused by CRAB treated with and without add-on tigecycline in combination regimena

|  | Original Cohort | |  | PS-matched cohort | |  |
| --- | --- | --- | --- | --- | --- | --- |
|  | With add-on  tigecycline | Without add-on  tigecycline | *P* value | With add-on  tigecycline | Without add-on  tigecycline | *P* value |
| **Case number** | 128 | 193 |  | 89 | 89 |  |
| Clinical failure |  |  |  |  |  |  |
| Day 7 | 59 (46.1%) | 85 (44.0%) | 0.717 | 43 (48.3%) | 39 (43.8%) | 0.548 |
| Day 14 | 52 (40.6%) | 101 (52.3%) | 0.040 | 39 (43.8%) | 53 (59.6%) | 0.036 |
| Day 28 | 56 (43.8%) | 99 (51.3%) | 0.185 | 40 (44.9%) | 54 (60.7%) | 0.036 |
| All-cause mortality |  |  |  |  |  |  |
| Day 28 | 34 (26.6%) | 71 (36.8%) | 0.056 | 25 (28.1%) | 38 (42.7%) | 0.042 |
| Hospital mortality | 65 (50.8%) | 91 (47.2%) | 0.524 | 43 (48.3%) | 69 (77.5%) | <0.001 |
| 28-day ventilator weaning^b^ | 62/125 (49.6%) | 79/172 (45.9%) | 0.532 | 45/87 (51.7%) | 32/83 (38.6%) | 0.085 |
| Newly onset dialysis^c^ | 9 (7.0%) | 20 (10.4%) | 0.519 | 6 (6.7%) | 13 (14.6%) | 0.089 |
| ICU stays | 27 (17-44) | 23 (16-40) | 0.093 | 27 (18-46) | 23 (16-40) | 0.454 |
| Hospital stays | 51 (36-76) | 46 (29-68) | 0.062 | 52 (39-80) | 45.5 (29-73) | 0.118 |

^a^Data are presented as n (%)

^b^Only cases with invasive ventilator were included for analysis

^c^Including hemodialysis and continuous venovenous hemofiltration within 28 days

CRAB, carbapenem-resistant *Acinetobacter baumannii* complex
